# Significance estimation for large scale metabolomics annotations by spectral matching

**DOI:** 10.1038/s41467-017-01318-5

**Published:** 2017-11-14

**Authors:** Kerstin Scheubert, Franziska Hufsky, Daniel Petras, Mingxun Wang, Louis-Félix Nothias, Kai Dührkop, Nuno Bandeira, Pieter C. Dorrestein, Sebastian Böcker

**Affiliations:** 1 Chair for Bioinformatics, Friedrich Schiller University Jena, Jena, 07743 Germany; 2 RNA Bioinformatics and High Throughput Analysis, Friedrich Schiller University Jena, Jena, 07743 Germany; 3 Collaborative Mass Spectrometry Innovation Center, Skaggs School of Pharmacy and Pharmaceutical Sciences, University of California, La Jolla, San Diego, CA 92093 USA; 4 Skaggs School of Pharmacy and Pharmaceutical Sciences, University of California, La Jolla, San Diego, CA 92093 USA

## Abstract

The annotation of small molecules in untargeted mass spectrometry relies on the matching of fragment spectra to reference library spectra. While various spectrum-spectrum match scores exist, the field lacks statistical methods for estimating the false discovery rates (FDR) of these annotations. We present empirical Bayes and target-decoy based methods to estimate the false discovery rate (FDR) for 70 public metabolomics data sets. We show that the spectral matching settings need to be adjusted for each project. By adjusting the scoring parameters and thresholds, the number of annotations rose, on average, by +139% (ranging from −92 up to +5705%) when compared with a default parameter set available at GNPS. The FDR estimation methods presented will enable a user to assess the scoring criteria for large scale analysis of mass spectrometry based metabolomics data that has been essential in the advancement of proteomics, transcriptomics, and genomics science.

## Abstract (web summary)

Matching fragment spectra to reference library spectra is an important procedure for annotating small molecules in untargeted mass spectrometry based metabolomics studies. Here, the authors develop strategies to estimate false discovery rates (FDR) by empirical Bayes and target-decoy based methods which enable a user to define the scoring criteria for spectral matching.

## Introduction

Untargeted mass spectrometric (MS) analysis of small molecules is important in our understanding of (bio)chemical processes in the environment, ocean, and individual organisms^1–7^. In untargeted mass spectrometry experiments, tandem MS (MS/MS) spectra are collected of molecules present in the analytical sample. To annotate these unknowns, the MS/MS spectra are compared against a library of reference MS/MS spectra^8–11^. At present, spectrum–spectrum matches of unknown and library spectra are scored but this score alone provides no statement about statistical accuracy of that assignment. Without statistical techniques in place to estimate false discovery rates of identifications, researchers do not have a guide to set appropriate scoring criteria, unlike in proteomics, peptidic small molecule identification, transcriptomics, and genomics where statistical assessment and false discovery calculations for annotations are the norm^12–15^. In metabolomics, probabilistic assignments of molecular formulas has been done but this does not provide structure identifications which are critical to biological understanding^16, 17^. This leads untargeted liquid chromatography tandem mass spectrometry (LC–MS/MS) based metabolomics or any other small molecule based untargeted mass spectrometry analysis to yield identification results where errors rates are uncontrolled. This can lead to a lack of sensitivity or worse: rampant false discoveries and ultimately incorrect interpretations.

To compound the challenge, due to advances in instrumentation and the re-emergence of appreciation in the function of small molecules, the scientific community is producing more and more untargeted mass spectrometry data. These LC–MS/MS based experiments are now commonly applied in medicine, life science, agriculture toxicology, exposomics, ocean and forensic research to name a few. Modern instruments generated hundreds to thousands of MS/MS spectra from a single sample,and collectively tens of millions of MS/MS spectra for large scale projects. There are also a growing number of MS/MS spectra available in public spectral libraries^8, 9, 11, 18, 19^. To most in the scientific community, including mass spectrometrists and metabolomics investigators themselves, it often comes as a surprise that there is no significance estimation in metabolomics annotations yet, like it has been adopted, and thereby advanced, in the fields of proteomics, genomics and transcriptomics. While guidelines and rules have been established by the metabolomics standards initiative^20^, to report annotation of molecules from MS-based metabolomics data^20^, they are not commonly reported in the majority of metabolomics studies. Manual validation at the scale of tens of thousands to millions of spectra library matches is not realistic to do for each large scale experiment, and automated solutions for the annotations that enable downstream analysis such as pathway mapping, xenobiotic metabolism, chemical ecology, and ultimately prioritization for manual validation are needed; but this process starts with annotations^21^.

Large scale non-targeted LC–MS/MS experiments result in hundreds to thousands of query spectra from a single chromatographic run. For molecular annotation, these MS/MS spectra are typically searched against a spectral library, which in turn, results in spectral library hits that are sorted by score. Using a decoy spectral library to estimate FDR is common in proteomics; there, the decoy database is often a (pseudo-)reverse peptide database or a shuffled database^12, 22, 23^. The reason why target-decoy approaches for FDR estimation have not been applied so far to metabolomics, are the difficulties in generating decoy libraries; small molecules are diverse in structure, and shuffling or reversing a database is not possible. Therefore, alternative strategies needed to be developed for FDR estimation. Here we present and assessed four possible solutions.Ultimately we implemented one method, the re-rooted fragmentation tree, in the MS/MS data analysis platform Global Natural Product Social Molecular Networking (GNPS, http://gnps.ucsd.edu)^9^, to demonstrate that FDR estimations need to be used to guide scoring parameters. To validate the FDR approach and how it performs for spectral annotation with real large scale untargeted mass spectrometry data, we performed FDR controlled spectral library matching with 70 data sets from GNPS, consisting of thousands of LC–MS runs. This revealed that there is no universal scoring criteria that can control the FDR in all data sets. This adaptive approach shows promise to both, increase identifications and curb false positives in large scale metabolomics experiments. The FDR estimation has now been implemented as a tool called passatutto, named after a food mill used to remove unwanted particles commonly used in Italian kitchens. Ultimately, passatutto provides experimentalists with an high-throughput measure of confidence in MS/MS-based annotations by reporting an FDR, to guide the selection of scoring parameters for a project compatible with large scale MS/MS based untargeted metabolomics projects.

## Results

### Construction of FDR estimation approaches

**Fig. 1:**
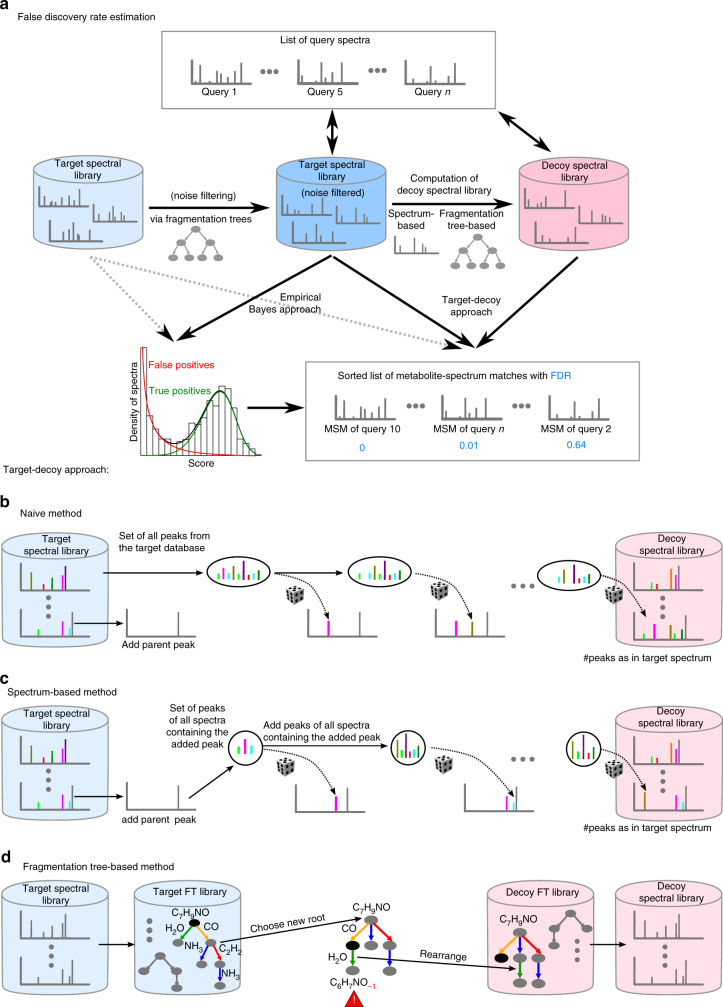
False discovery rate estimation. **a** Overview. The empirical Bayes approach estimates FDRs from a two-component mixture of distributions representing true and false hits (positive identifications). In the target-decoy approach, query spectra are searched against a target and decoy spectral library, and FDRs are estimated from the merged and sorted list of spectrum matches. **b**–**d** To construct a decoy spectral library, we implemented three methods. **b** Naive method: randomly adding fragment ions from the reference library to the decoy spectrum. **c** Spectrum-based method: fragment ions are iteratively added to the decoy spectrum, conditional on fragment ions that have previously been added. **d** Fragmentation tree-based method: a fragmentation tree is computed from the target spectrum, its root is relocated. New formulas of fragments are calculated according to the losses in the tree. Fragments with invalid formulas are relocated

Our first method that we assessed uses an empirical Bayes approach^24^ whereas the second, third and fourth FDR estimation methods rely on the target-decoy approach, using different decoy databases (Fig. 1a–d). Although the generation of “random” MS/MS spectra for small molecules is conceptually more challenging than for peptides^25^, it became possible with recent methodological advances^9, 26–28^. To estimate the FDR using a decoy database, three strategies were devised to create the decoy MS/MS library (Fig. 1b–d), where the first two methods are spectrum-based while the third is fragmentation tree-based^23, 24^. To show compatibility with different spectral matching scoring schemes, we present results for the MassBank scoring^11^ and the GNPS scoring^9, 29^, both of which utilize modified versions of the cosine similarity (also known as normalized dot product).

For the naive decoy spectral library, we use all possible fragment ions from the reference library of spectra and then randomly add these ions to the decoy spectral library, until each decoy spectrum reaches the desired number of fragment ions that mimics the corresponding library spectrum (Fig. 1b). This method is presented as a baseline evaluation of the other, more intricate methods. The second method is similar to the naive method, as we create the decoy spectral library through choosing fragment ions that co-appear in the spectra from the target spectral library (Fig. 1c): In this spectrum-based approach, we start with an empty set of fragment ion candidates. First, the precursor fragment ion of the target spectrum is added to the decoy spectrum. For each fragment ion added to the decoy spectrum, we choose all spectra from the target spectral library which contain this fragment ion, within a mass range of 5 p.p.m. From these spectra, we uniformly draw (all fragment ions have the same probability to be drawn) five fragment ions that are added to the fragment ion candidate set; we use all fragment ions in case there are fewer than five. We draw a fragment ion from the fragment ion candidate set and add it to the decoy spectrum, then proceed as described above until we reach the desired number of fragment ions that mimics the corresponding library spectrum. The two-step process of first drawing candidates, then drawing the actual decoy spectrum was introduced to better mimic fragmentation cascades and dependencies between fragments. Furthermore, it prevents that fragment-rich spectra dominate the process. Out of the five added candidate fragment ions, between zero and five end up in the final decoy spectrum. Fragment ions with mass close (5 p.p.m.) to a previously added fragment ion mass, or masses above the precursor fragment ion mass are discarded. If the precursor ion is absent from the MS/MS spectrum, we use the selected ion mass to find matching compound masses. The third solution is a fragmentation tree-based approach, where decoy spectra are generated using a re-rooted fragmentation tree (Fig. 1d). From the original fragmentation tree, its structure and all losses are kept, and some new internal node is selected as new root, with the molecular formula of the precursor ion. Molecular formulas of all fragment ions are calculated along the edges of the tree, subtracting losses. In case the tree rearrangement yields chemically impossible molecular formulas (that is, a negative number of atoms for some element), the corresponding loss and its subtree are placed to another branch of the tree (re-grafted), attaching it to a uniformly selected node. The new root node is not drawn uniformly: Instead, a node is chosen as new root with relative probability 1/(*n*+1), where *n* is the number of edges that we would have to re-graft. For all three methods, intensities of the original fragment ions are used.

### Assessing quality against spectral libraries

**Fig. 2:**
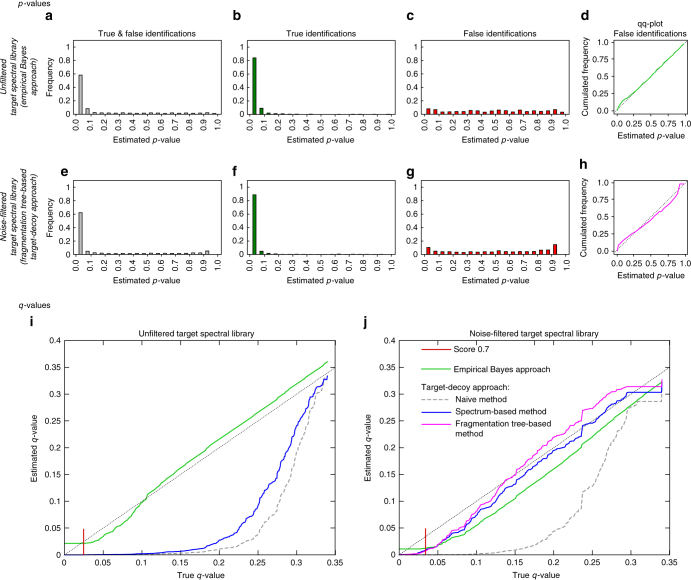
Quality assessment for FDR estimations for Agilent query spectra to the GNPS library using MassBank scoring function. **a**–**h** *p*-values. Distribution of *p*-values. For searching in the unfiltered target spectral library **a**–**d**, *p*-values are estimated using the empirical Bayes approach. For searching the noise-filtered target spectral library, *p*-values are estimated using the fragmentation tree-based target-decoy approach **e**–**h**. Distributions contain *p*-values from ten decoy spectral libraries. *p*-value distribution for both, true and false hits **a**, **e**, *p*-value distribution for true hits only **b**, **f**, and for false hits only **c**, **g**. By definition, the distribution of *p*-values for false hits has to be uniform, corresponding to the main diagonal in the *p*-value quantile-quantile (qq) plots **d**, **h**. The qq plots for the other methods are provided as Supplementary Fig. 1. **i**, **j** *q*-value plots for Agilent data (*q*-value plots for MassBank are provided as Supplementary Fig. 2). Estimated (*y*-axis) vs. true *q*-values (*x*-axis) in the unfiltered **i** and noise-filtered **j** version of the GNPS library. The small red line indicates cosine of 0.7. For the fragmentation tree-based method, we searched against the noise-filtered GNPS only, since this approach applies noise-filtering by design. The naive target-decoy approach can be seen as baseline method for comparison. For target-decoy methods, results were averaged over ten decoy spectral libraries (Supplementary Fig. 4)

Assessing the quality of empirical Bayes, and the naive, spectrum-based and fragmentation tree-based target decoy databases was done by *p*-value estimation, and by testing *q*-value estimates against exact values using public MS/MS libraries. Evaluation can only be carried out when the true identity of all query compounds is known. To assess quality, we used high resolution reference spectra from the Agilent^30^, MassBank^11^, and GNPS libraries^9^. From GNPS and MassBank, only spectra that had the unfiltered spectrum in the public domain, that had SMILES or InChI structure annotations (line notations for describing chemical structure using short strings) and for which the precursor mass matched to the exact structure-based mass to within 10 p.p.m., were used for the assessment of the FDR estimations. As an initial test, we checked if *p*-values of false hits (false positive identifications) estimated by our methods are uniformly distributed^31^: The *p*-value of a spectrum match is the probability to randomly draw a result of this or better quality, under the null hypothesis for which a spectrum has been randomly generated. We observe a mostly uniform distribution of *p*-values, both for the empirical Bayes approach and the fragmentation tree-based target-decoy approach (Fig. 2a–f), corresponding to a quantile-quantile plot close to the main diagonal (Fig. 2d, h, Supplementary Fig. 1). This agrees with the distribution of *p*-values under the null hypothesis, and shows that our decoy databases are indeed representative models of the null hypothesis. In the *p*-value distribution, we observe heightened peaks close to 0 and 1; the heightened peak close to 0 is discussed below, whereas the heightened peak close to 1 is negligible for significance estimation.

To evaluate the quality of estimated FDRs, we compared *q*-values of the four methods presented here with true *q*-values. In addition, we also assessed the impact of noise filtering on the quality of FDR estimation: Noise-filtering by fragmentation trees is accomplished by calculating a fragmentation tree that annotates some of the hypothetical fragment ions with molecular formulas^32, 33^; only these annotated fragment ions are kept, resulting in a cleaned spectrum that only keeps fragment ions that are well-supported by the fragmentation process. For the unfiltered target spectral library, empirical Bayes approach resulted in good estimates, whereas spectrum-based target decoy did not work as accurately (Fig. 2g, h): the empirical Bayes approach represented a good fit of the bisecting line, while the spectrum-based approach did not. For the noise-filtered target spectral library, the target-decoy methods except the naive method allow for accurate *q*-value estimates, and perform roughly on par (Fig. 2c). The naive method never results in accurate *q*-value estimates: Even for true *q*-values around 0.15, estimates are already close to 0. All methods tend to overestimate significance (estimated *q*-values are smaller than true *q*-values); in particular, estimates are close to zero for true *q*-values below 0.05. For some query compounds, not contained in the target database, there is a structurally similar isomer with similar fragmentation spectrum present in the target database (Supplementary Fig. 2). These wrong hits will receive relatively high scores and, hence, wrong hits in the target database are more frequent at top positions of the output list than hits in the decoy database, impeding accurate estimation for small *q*-values.

Results in Fig. 2g, h are for Agilent queries; see Supplementary Figure 3 for MassBank queries. To further evaluate the robustness of our estimates, we generate 10 decoy spectral libraries for each decoy method. Because generating decoy spectral libraries is a random process, *q*-values vary slightly between the 10 decoy spectral libraries; we found these variations to be negligible (Supplementary Fig. 4). Results in Fig. 2 present searching Q-TOF spectra using the MassBank scoring function. Results for the cosine similarity score were comparable (Supplementary Fig. 5). Furthermore, using Orbitrap MassBank spectra as queries yielded similar results (Supplementary Fig. 2).

### Evaluation of fragmentation tree decoy strategy against public data

**Fig. 3:**
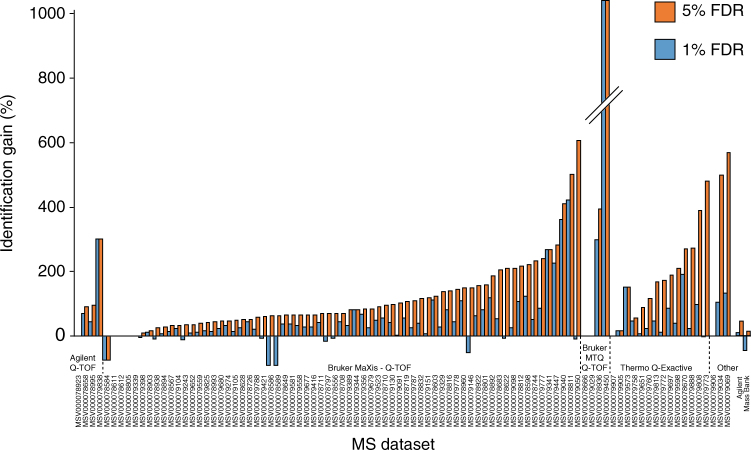
FDR based annotations for 70 metabolomics projects. These are projects from human, microbes, plants, marine-organism, and other derived metabolomics data. The plot shows the percent gain in annotations for each of the data sets in GNPS-MassIVE at 1% and 5% FDR in relationship to the mass spectrometer used. A plot sorted by sample characteristic is provided as Supplementary Fig. 7. The impact on identification rates with the MassBank and Agilent data sets are shown on the right

We evaluated the fragmentation tree-based decoy FDR estimation method broadly across 70 data sets available on GNPS. We selected a decoy-based FDR estimation, as this does not rely on presupposed underlying (and potentially unrelated) probabilistic models^34^. We also choose the filtered decoy approach as this is compatible with the filtered data in GNPS. Data sets included high resolution Q-TOF or Orbitrap data from 6220 LC-MS runs encompassing human, microbe, plant and marine-organism derived samples. To calculate both the 1% FDR and 5% FDR, the total running time for the FDR computation of the spectral library matches associated with all the projects took ~48 h on the GNPS cluster, demonstrating the compatibility of the FDR approach with large-scale metabolomics experiments. At 1% FDR, the average gain in annotation for the 70 public data sets in comparison to default scoring cut-off value (cosine score of 0.7 and minimum of 6 ions to match) was 139% with a range of −92 up to 5705% (Fig. 3, Supplementary Figs. 6 and 7). At a score of 0.7, the annotations from continuous identification, as judged by the community via a four-star rating of the identifications, the GNPS community provided feedback that 91% of the annotations are correct, 4% possible isomers or correct, 4% not enough information to tell and 1% is incorrect^9^. When using 5% FDR, a mean gain annotation of 235% was obtained and had a range of −75% up to 6705% gain (Fig. 3).

**Fig. 4:**
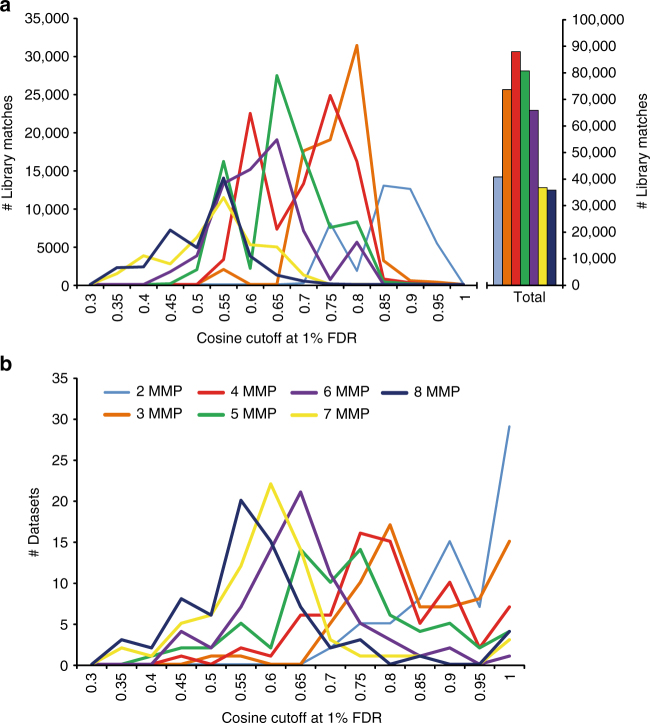
The impact of number of matching fragment ions in a spectrum and cosine score at 1% FDR. **a** Frequency of data sets in relationship to number of MMP to match and cosine at 1% FDR estimation. **b** The number of MS/MS matches in relation to minimum matched fragment ions and cosine. An alternative 3D plot of these figures can be found in Supplementary Fig. 9

Further, we explore the impact of cosine scoring and the minimum number of fragment ions to match on the number of matches associated with 1% FDR using the fragmentation tree-based decoy strategy. Over the 70 public metabolomics projects, the minimum matched peaks were modulated resulting in a cosine threshold ranging from 0.3 to 1 with the number of identifications represented in a histogram (Fig. 4a). The results reveal that the more ions were required to match, the more forgiving the spectral scoring could be. When 8 ions were required to match, the most common score to achieve 1% FDR was found to be between a cosine of 0.50–0.60, while when two ions were required to match, the most common score required was 0.85–0.95. For all the projects that require a cosine of 1 to achieve 1% FDR, not a single annotation was obtained (Fig. 4b). We observed that the most number of annotations was achieved with a minimum of 4 fragment ions matching, with 3 ions and 5 ions as close second and third in terms of the number of spectra that were annotated. As the number of fragment ions required to match the number of matches was increased to 7 and 8 or lowered to 2, the number of total matches decreased significantly.

This comparison furthermore revealed that for most of the projects, an FDR of 1% was achieved at cosine of 0.6–0.65 (for 5% FDR, most of the projects dropped to a cosine of 0.5–0.55) and therefore the default cosine values for living data in GNPS are slightly more stringent. Three representative FDR vs. default score threshold plots are provided as Supplementary Fig. 8, and may guide the end user to make a decision on scoring criteria. At 1% FDR, it was observed that parameters of the GNPS living continuous annotation overestimates the number of annotations for 13% of the projects and that it underestimated the annotations in 82% of the projects and the remaining 5% of projects remained unchanged by the introduction of the FDR estimation.

## Discussion

The four methods that were implemented and assessed for significance of annotations for untargeted mass spectrometry are using empirical Bayes approach, which implies a probabilistic model of score distributions, and three different target-decoy approaches. Different from in silico annotation of MS/MS spectra of peptidic small molecules^35^, three decoy methods for building decoy spectral libraries that were formulated and tested do not generate artificial metabolite structures: both, the construction of metabolite structures which are plausible but non-existing in nature, and the prediction of fragmentation spectra from metabolites structures are extremely challenging problems and therefore we did not pursue those routes for significance estimation^35, 36^. To avoid this challenge, the decoy libraries are generated via the naive method, a spectrum-based and a fragmentation tree-based method. Using three test reference databases, Agilent^30^, MassBank^11^, and GNPS^9^, with thousands of MS/MS spectra that have the structures of molecules associated with them, we show that all but the naive methods can estimate false discovery rates^37^ (FDR, the proportion of false discoveries among the discoveries) and *q*-values (the minimal FDR thresholds at which given discoveries should be accepted) with high accuracy.

The key considerations that went into the design of the decoy spectral libraries was to ensure that decoy spectra mimic real spectra as closely as possible, but at the same time, do not correspond to MS/MS spectra of any true metabolites present in the sample. This ensures that hits in the decoy database are equally likely as false hits in the spectral library (the target database). In addition, we assured that for any precursor mass range, the same number of target and decoy spectra were found. All methods circumvent generating decoy structures, as it is unsolved problem to generate molecular structures which are sufficiently similar to the structures in the target spectral library, but not present in the sample. Generating decoy MS/MS spectra completely at random, i.e., randomly drawing both masses and intensities of the fragment ions, will not result in an adequate decoy spectral library, as there are ion masses that can be generated but will never be found in a real MS/MS spectrum. Addition of adducts to the spectra that are not encountered would be a solution to create a decoy spectral library, as it was recently done for precursor mass FDR calculations in imaging mass spectrometry annotation^32^; but these adducts would not look like spectra that we would encounter in an MS/MS spectrum from a biological sample and therefore this solution is not appropriate for the annotation of MS/MS spectra.

We further showed that the approach could be applied to different dot product like scoring methods (Supplementary Fig. 5) and therefore we anticipate that these methods can be used for other commonly used scoring schemes for spectral matching, such as cosine similarity itself^28, 38^, scorings based on the number of matching fragment ions and the sum of intensity differences^39^ or scorings which incorporate mass differences^40^.

It is evident that the methods are versatile but there are some limitations associated with significance estimations of spectral matches, some of these have been well documented for proteomics. All methods tend to overestimate significance (estimated *q*-values are smaller than true *q*-values); in particular, estimates are close to zero for true *q*-values below 0.05. For some query compounds not contained in the target database, there is a structurally similar isomer with similar fragmentation spectrum present in the target database (Supplementary Fig. 2). These wrong hits will receive relatively high scores and, hence, wrong hits in the target database are more frequent at top positions of the output list than hits in the decoy database, impeding accurate estimation for small *q*-values. This situation is different from shotgun proteomics for a single organism but similar to metaproteomics, where estimation of accurate *q*-values close to zero is equally challenging^41^. One of the key reasons is that some small molecules, in particular isomeric structures (Supplementary Fig. 2), have nearly identical MS/MS spectra. Under such circumstances the end user would have to consider all isomers and perform follow up experiments to differentiate among the possibilities.

Because of the probabilistic model requirements for Empirical Bayes and the observation the fragmentation tree decoy strategy performed best of all the decoy strategies with filtered data, the type of data that is most readily accessible in GNPS. In addition, empirical Bayes allows us to estimate Posterior Error Probabilities; but the method relies on presupposed probabilistic models, whereas target-decoy approaches make no further modeling assumptions^34^. To avoid making assumptions of a model required by Empirical Bayes and because the fragmentation tree decoy strategy was the most compatible with the data type in GNPS, it was implemented as FDR controlled spectrum matching workflow into GNPS. Passatutto was tested against 70 mass spectrometry projects in the public domain The results show that the same spectrum matching score can contribute to a highly variable FDR and that the FDR can be drastically different for each project. This means that the spectral scoring for annotations needs to be adjusted on a per project basis and based on the false discovery rates the end user is willing to accept. With the 70 projects analyzed there were no trends with respect to the instrument type observed, in agreement with our benchmark results (Fig. 2).

However, notable trends were observed in relation to number of fragment ions used to match MS/MS spectra. As the number of fragment ions required to match the number of matches dropped to 3 and 2, the number of total matches decreased significantly. At these scores, there is not enough spectral information to differentiate a match to the library from the decoy library and therefore drives up the FDR more quickly. However, there is a clear optimum at 4, 5, 6 ions because as it requires 7 and 8 ions to match, a decrease in the number of annotations can be observed. This was explained by the fact that fewer spectra havinge a minimum of seven or eight fragment ions were able to match.

We can further compare those results to the results from the default GNPS scoring value of cosine of >0.7 and a minimum of 6 fragment ions to match^9^ (If multiple reference spectra exist that satisfy these criteria, only the best-scoring reference is used as a “hit”). The GNPS community assessed matches are the only direct comparisons that can be used to asses how the FDR estimation impacts results of large scale spectrum library matching.However, a key observation is that for each data set the cosine scoring needs to be adjusted when compared to the default GNPS parameters of cosine of 0.7 or greater and minimum 6 fragment ions. In other words, GNPS living data enabled through continuous annotation^9^ that uses just one specific scoring value not only underestimates the annotations for most projects but perhaps more importantly, as this affects the interpretation of the results, living data also overestimates the number of annotations for some projects.

The reason why most GNPS projects analyzed with living data parameters underestimate the number of matches is because there are many molecules that do not provide 6 ions when fragmented. These are currently missed by living data in GNPS. Thus, FDR calculations enable an informed decision in terms of the analysis parameters that a researcher can use in terms of deciding what the level of acceptable incorrect annotations that can be expected with such parameters. These results demonstrated why the introduction of significance estimation and FDR assessments are critical for the field of untargeted small molecule mass spectrometry and that significance estimations needs to become a routine part of this field.

In summary, all tested methods but the naive allow for FDR estimation. The decoy-based methods do not rely on presupposed underlying probabilistic models, but require that target mass spectra are noise-filtered to reach accurate estimates. We use fragmentation trees^26, 27, 33^ to separate signal fragment ions from noise fragment ions to “clean” target spectra for the generation of decoy libraries. We demonstrated that this approach can be used for providing confidence measures in large scale metabolomics project, where it is becoming more and more impossible to inspect each annotation by hand, which is the current norm in metabolomics. It revealed that the spectrum scoring parameters need to be adjusted on a per-project-basis, which requires a form of confidence measures associated with the results. As such evaluations have been critically important for advancing other fields such as proteomics, genomics and other fields, we anticipate that this will play a similarly critical role with mass spectrometric analysis of small molecules in the future. In that perspective, we integrated passatutto into GNPS web platform to ensure that the community can readily search spectral libraries in high-throughput manner while reporting a significance of the annotation. Our methods constitute the first step towards FDR estimation of annotations in untargeted metabolomics, but we anticipate that additional advances will be made in the years to come. We further envision that robust accuracy estimations, including FDR, will also enhance the analysis of spectral matches for in silico generated reference libraries or in silico annotations^4, 42–47^, that^48^ are beginning to play important roles in brightening the dark matter of untargeted metabolomics ^4, 49–51^.

## Methods

### Dataset

All metabolomics data used was from GNPS http://gnps.ucsd.edu

The accession numbers for the data sets used are MSV000078567, MSV000078584, MSV000078586, MSV000078589, MSV000078598, MSV000078603, MSV000078611, MSV000078612, MSV000078628, MSV000078649, MSV000078658, MSV000078670, MSV000078683, MSV000078708, MSV000078710, MSV000078711, MSV000078719, MSV000078726, MSV000078744, MSV000078805, MSV000078811, MSV000078812, MSV000078816, MSV000078832, MSV000078892, MSV000078903, MSV000078922, MSV000078936, MSV000078960, MSV000078993, MSV000079029, MSV000079040, MSV000079050, MSV000079069, MSV000079091, MSV000079104, MSV000079105, MSV000079146, MSV000079243, MSV000079329, MSV000079339, MSV000079341, MSV000079344, MSV000079356, MSV000079398, MSV000079416, MSV000079421, MSV000079447, MSV000079450, MSV000079558, MSV000079573, MSV000079581, MSV000079598, MSV000079651, MSV000079652, MSV000079679, MSV000079758, MSV000079760, MSV000079772, MSV000079773, MSV000079777, MSV000079778, MSV000079787, MSV000079808, MSV000079813, MSV000079825, MSV000079838, MSV000079888, MSV000079905, MSV000079907.

### Spectral libraries and processing

We use three reference libraries for evaluating our FDR estimations: Agilent, MassBank and GNPS. The requirements for a MS/MS spectrum of a compound to be included in the analysis are that they had to (a) have a SMILES or InChI associated with it; (b) to remove low resolution reference data, the exact precursor mass must be within 10 p.p.m. of the observed mass; (c) the unfiltered MS/MS spectrum has to be available in the public domain. To ensure maximal homogeneity, we keep only (d) spectra in positive ion mode, (e) compounds below 1000 Da, and we discard (f) spectra with <5 peaks with relative intensity above 2%. Spectra recorded at different collision energies are merged. In total, MS/MS spectra of 6716 compounds (4138 GNPS, 2120 Agilent, 458 MassBank) fit these criteria. Most GNPS and all Agilent spectra were measured on Q-TOF instruments, all MassBank spectra were recorded on Orbitrap instruments. Not all peaks/signals in an MS/MS spectrum can be explained as fragment ions^32^, but we will stick with the term “fragment ion” instead of “hypothetical fragment ion”, “peak” or “signal” for the sake of readability. Similarly, we will speak of the “mass” of a fragment ion when we refer to the observed *m/z* value, and of its ‘intensity’ when we refer to the peak intensity.

### Noise filtering

For each target MS/MS spectrum, we calculate a fragmentation tree that annotates a subset of hypothetical fragment ions with molecular formulas^27, 32, 33^; only annotated fragment ions are kept, using the original peak intensities. We set mass deviation parameters 10 p.p.m. (relative) and 2 mDa (absolute). This procedure is more sensitive than simply using a hard or soft intensity cutoff, as it ensures that fragment ions can be explained in principle by some sensible fragmentation cascade. After noise filtering, 104 spectra were empty or consisted only of the precursor ion peak, and were discarded.

### Software and creation of decoy databases

Passatutto has been implemented as a Java v1.6 program. It reads and writes spectra in MassBank file format and fragmentation trees in the SIRIUS DOT file format. Source code is available from https://github.com/kaibioinfo/passatuto, Java executables (JAR files) are available from https://bio.informatik.uni-jena.de/passatutto/. Passatutto contains modules for (a) generating a decoy database, (b) database searching in locally stored data sets, and (c) estimating *q*-values either by means of the target-decoy approach or by empirical Bayes estimation. For generating a decoy database using the fragmentation tree-based method, SIRIUS can be used for the computation of fragmentation trees, which is available from https://bio.informatik.uni-jena.de/sirius/. We ran the software on an Intel XEON 6 Core E5-2630 at 2.30 GHz with 4 GB memory.

### FDR estimation

Given a decoy database, FDRs and *q*-values can be estimated using target-decoy competition^25^, separated target-decoy search^23^, or the more sophisticated mix-max approach^52^. Here, we use a simple separated target-decoy search^23^ where the proportion of incorrectly annotated spectra^31^ is estimated from the empirical Bayes distribution. Only the best-scoring reference spectrum is referred to as the “hit” in the target database, as this represents the most likely interpretation of the query data. We merge lists of hits from target and decoy database, and sort by score. For any score threshold, we only report hits above this threshold in the target database, and estimate the number of false hits there using the number of hits in the decoy database above the score threshold. We estimate the FDR as percentage of incorrect targets (PIT) times the number of decoy hits above score threshold, divided by the number of target hits above score threshold^23^. The PIT is the percentage of hits in the target database that are incorrect, when we do not apply a score threshold but consider the complete batch of queries. We estimate PIT as the area under curve for false identifications, using the empirical Bayes approach described in Supplementary Information. The *q*-values of a hit is the minimal FDR at which this hit is present in the output list, varying over all possible score thresholds.

For the empirical Bayes approach^26, 52^, we model database search scores as a two-component mixture of distributions representing true hits and false hits (true positive and false positive identifications). Scores of true hits are modeled using a mirrored Gamma distribution, a mirrored Gumbel distribution, or a mirrored Weibull distribution, whereas scores of false hits are modeled using a Gamma distribution. Both the actual distribution for true hits and the distributions’ parameters are chosen based on the observed data, where Expectation Maximization is used to simultaneously find the parameters of the mixture distribution. FDR and *q*-values are estimated using the average Posterior Error Probability of all hits with score above the threshold; the Posterior Error probability for a given score, in turn, is estimated as the proportion of incorrect hits among all hits with this score. See Supplementary Information.

### Quality assessment of FDR estimation

The two smaller data sets, MassBank and Agilent, are used as query spectra, whereas the larger GNPS dataset is searched in. The estimated *p*-value is the ratio of decoy hits with score above the threshold. As we know the true identity of all queries, we can calculate the true FDR (ratio of false hits among all hits) for any score threshold; by definition, the *q*-value of a hit is the smallest FDR for which it is reported.

### FDR based annotations for metabolomics

Passatutto produced decoy spectra for GNPS’s spectral library search workflows. These workflows were altered to enable FDR estimation utilizing these decoys to estimate provide *q*-values for all identifications in a search. The number of identifications were reported at 1% and 5% FDR for each of the 70 data sets analyzed in GNPS and were compared against the default scoring thresholds recommended at GNPS (0.7 cosine, 6 minimum matched peaks).

### Impact of scoring parameters that achieve 1% FDR

To evaluate how scoring settings such as cosine score and number of minimum ions to match affected the number of annotations with an FDR of 1%, we ran passatutto on the same 70 public projects but varies the number of minimum matched ions from 2 to 8. We then reported the number of data sets that achieved 1% FDR at each cosine value and minimum number of ions that matched. Finally, we reported the number of spectra matched for all of the different projects.

### Empirical Bayes Approach

For details of the empirical Bayes approach, please see Supplementary Methods.

### Data availability

Link to passatuto application and used spectral data:

https://bio.informatik.uni-jena.de/passatutto/

Github link to passatuto source code:

https://github.com/kaibioinfo/passatuto

Web based passatuto: http://gnps.ucsd.edu/ProteoSAFe/static/gnps-experimental.jspAll metabolomics datasets are listed in ‘Dataset’.

## Electronic supplementary material

Supplementary Information

Peer Review File
