# Supplementary material for: Significance estimation for large scale metabolomics annotations by spectral matching
Source: Nat Commun. 2017 Nov 14;8:1494. doi: 10.1038/s41467-017-01318-5 (PMC5684233; doi:10.1038/s41467-017-01318-5)
Supplement: Supplementary file 1 — Supplementary Information [file 41467_2017_1318_MOESM1_ESM.pdf]

Supplementary Figures

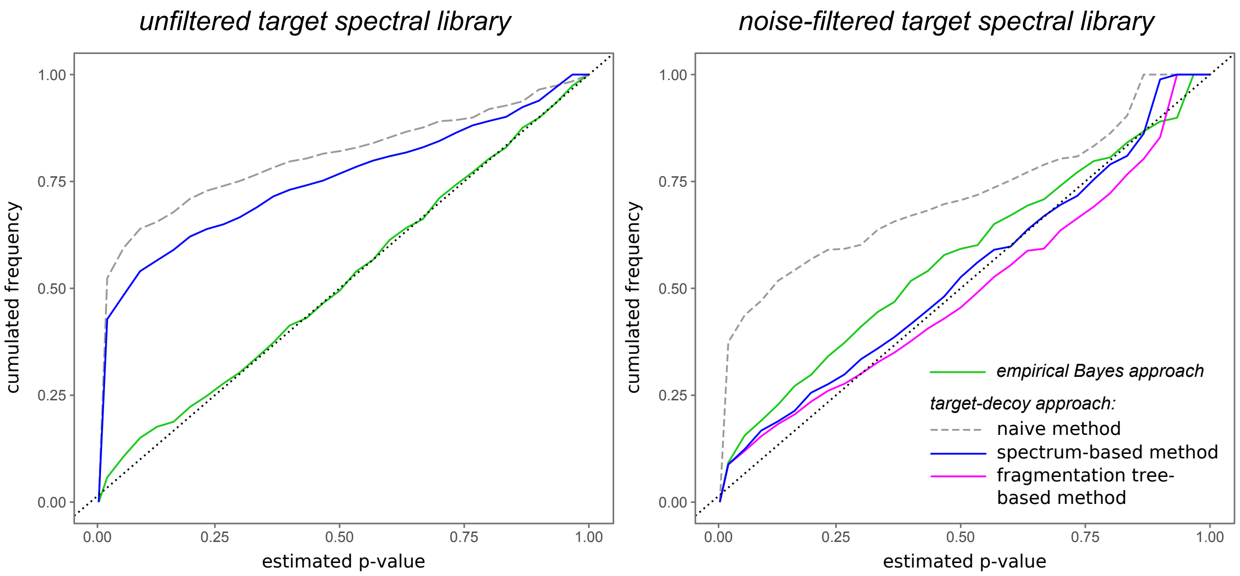

Supplementary Figure 1: **Quantile-quantile plots of estimated p-values** for false hits when searching for Agilent query spectra in the unfiltered (left) and noise-filtered (right) version of the GNPS library. p-values from ten decoy spectral libraries are used. By definition, the distribution of p-values for false hits has to be uniform, corresponding to the main diagonal in the quantile-quantile plots.

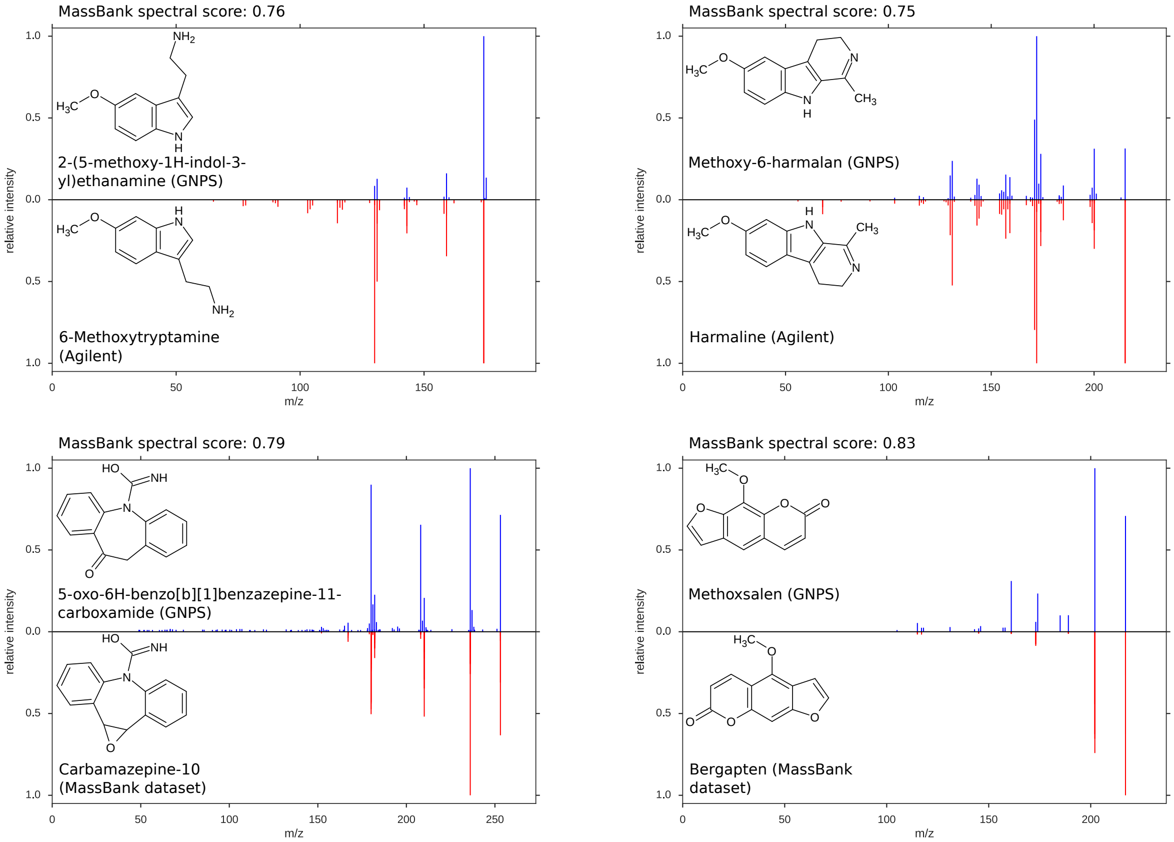

Supplementary Figure 2: Four examples of query compounds from Agilent and MassBank datasets that were absent from the target library GNPS. In all cases, we find an isomer in the target library that has high structural similarity to the query; in all cases, the spectral similarity between query and this hit in the target library is 0.75 or higher using the MassBank score, and would be accepted using a fixed threshold of 0.7. The Agilent dataset also contains Carbamazepine-10, and querying this spectrum results in the same hit in the GNPS library, with the same MassBank score of 0.79.

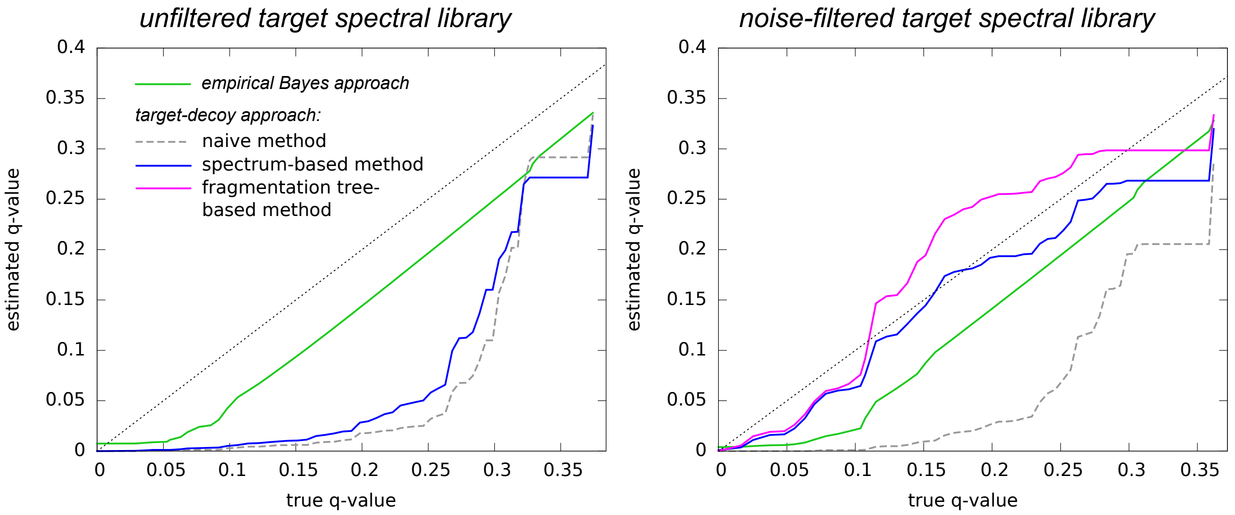

Supplementary Figure 3: Estimated (y-axis) vs. true q-values (x-axis) for searching **MassBank query spectra collected on a Orbitrap instrument** in the unfiltered (left) and noise-filtered (right) version of the GNPS library, using the MassBank scoring function. Results for the empirical Bayes approach and the three target-decoy approaches. For the fragmentation tree-based method, we searched against the noise-filtered GNPS only, since this approach applies noise-filtering by design. For target-decoy methods, results are averaged over ten decoy spectral libraries. The stronger "jumping behavior" of the curve can be attributed to the fact that the MassBank dataset is almost an order of magnitude smaller than the Agilent dataset.

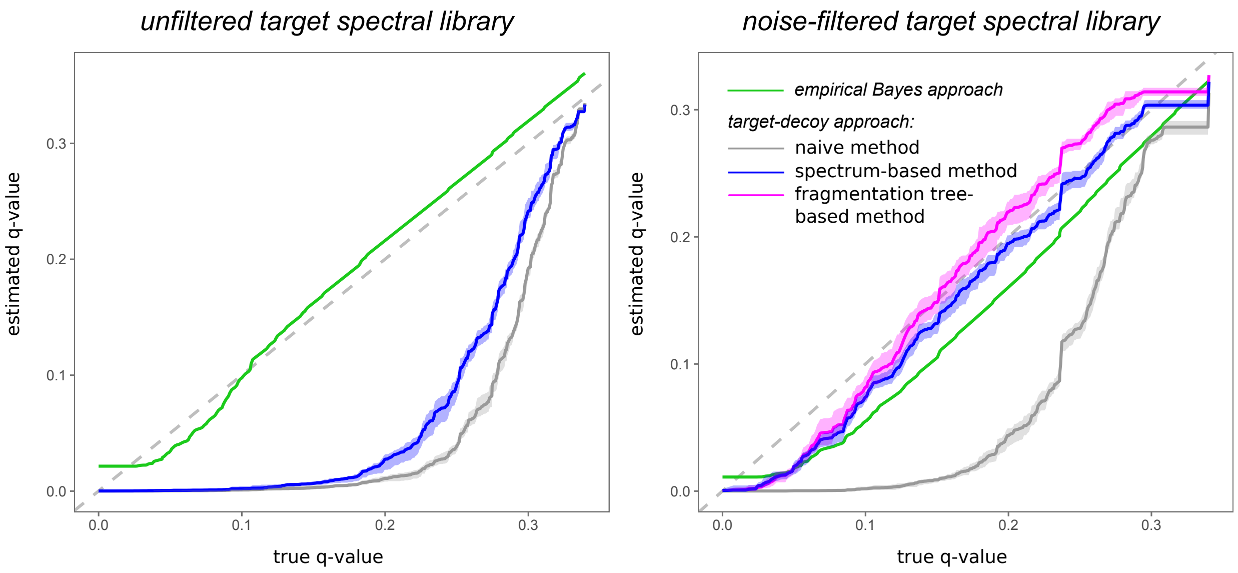

Supplementary Figure 4: Estimated (y-axis) vs. true q-values (x-axis) for searching Agilent query spectra mostly collected on a Q-tof instrument in the unfiltered (left) and noise-filtered (right) version of the GNPS library, using the MassBank scoring function and **ten independently created decoy databases**. The line shows the mean values for the ten decoy databases, whereas the range of estimated q-values is shaded. Results for the empirical Bayes approach and the three target-decoy approaches. For the fragmentation tree-based method, we searched against the noise-filtered GNPS only, since this approach applies noise-filtering by design.

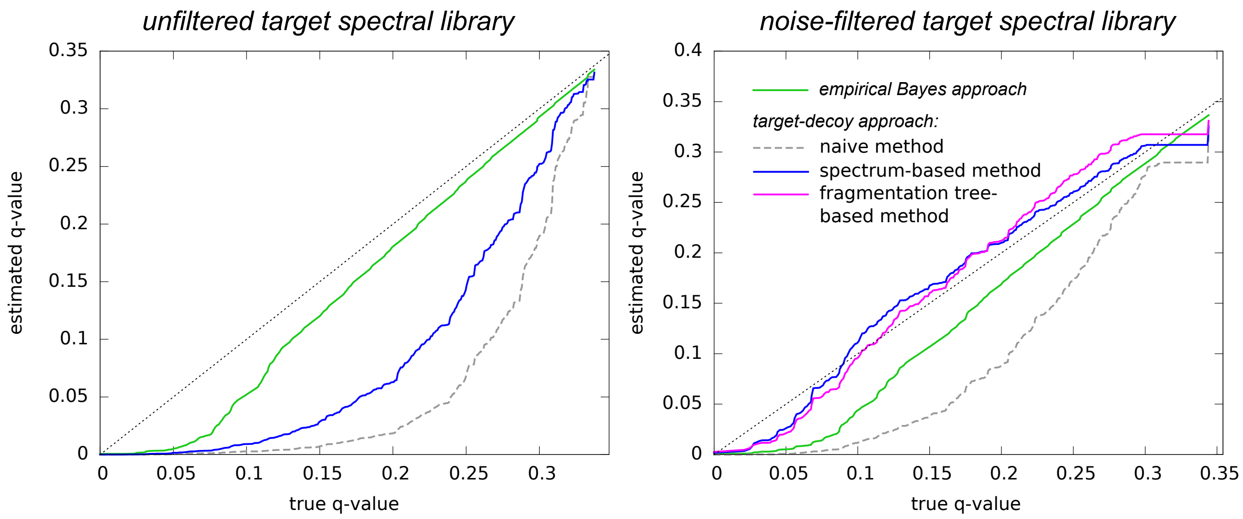

Supplementary Figure 5: Estimated (y-axis) vs. true q-values (x-axis) for searching Agilent query spectra mostly collected on a Q-tof instrument in the unfiltered (left) and noise-filtered (right) version of the GNPS library, using the **cosine scoring function**. Results for the empirical Bayes approach and the three target-decoy approaches. For the fragmentation tree-based method, we searched against the noise-filtered GNPS only, since this approach applies noise-filtering by design. For target-decoy methods, results are averaged over ten decoy spectral libraries.

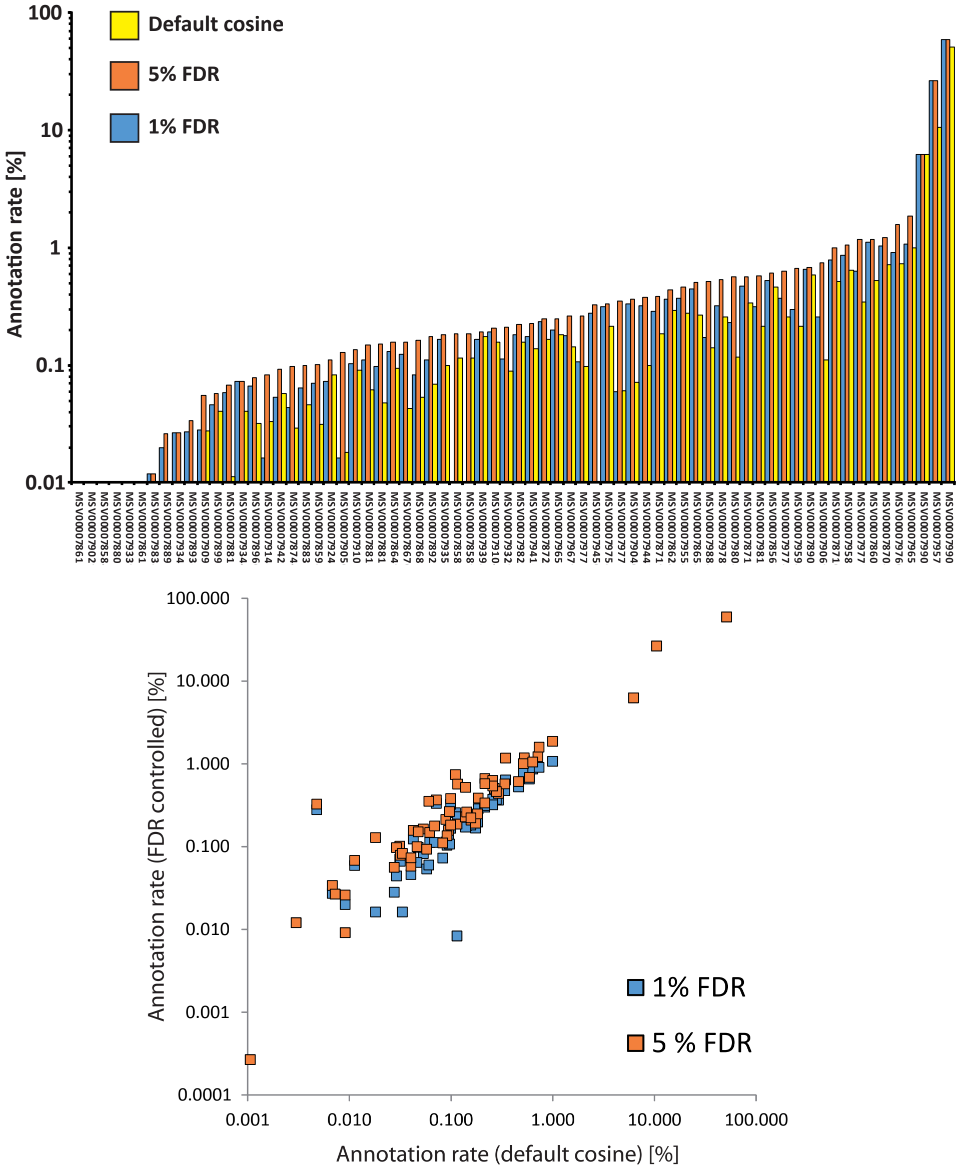

Supplementary Figure 6: Annotation rates. Top: Bar graph of annotation rates for each data set. Bottom: Annotation rate for 1% and 5% FDR cut-off in relation to the default cosine score of 0.7. The small annotation rates are due to a) less studied systems having few matches to molecules in public reference libraries, as there is a strong bias in them; and b) the limited reference spectra in the public domain that could be used for FDR estimations based on open access that allows redistribution and our inclusion criteria (see methods; we used the same spectra to determine the annotation rate at default GNPS settings) resulting in low resolution spectra not being included. This annotation value increases as the number of reference spectra that can be included and provided back to the community increase. Currently commercial licenses prevent the inclusion of larger libraries in the GNPS infrastructure and therefore this approach enables assessment of parameters to use for FDR calculations to use scoring parameters guided by statistics when performing a search with all the reference libraries accessible to the user. With MassBank, GNPS and other resources making this information freely available, the rate of annotations will increase when performing FDR estimations.

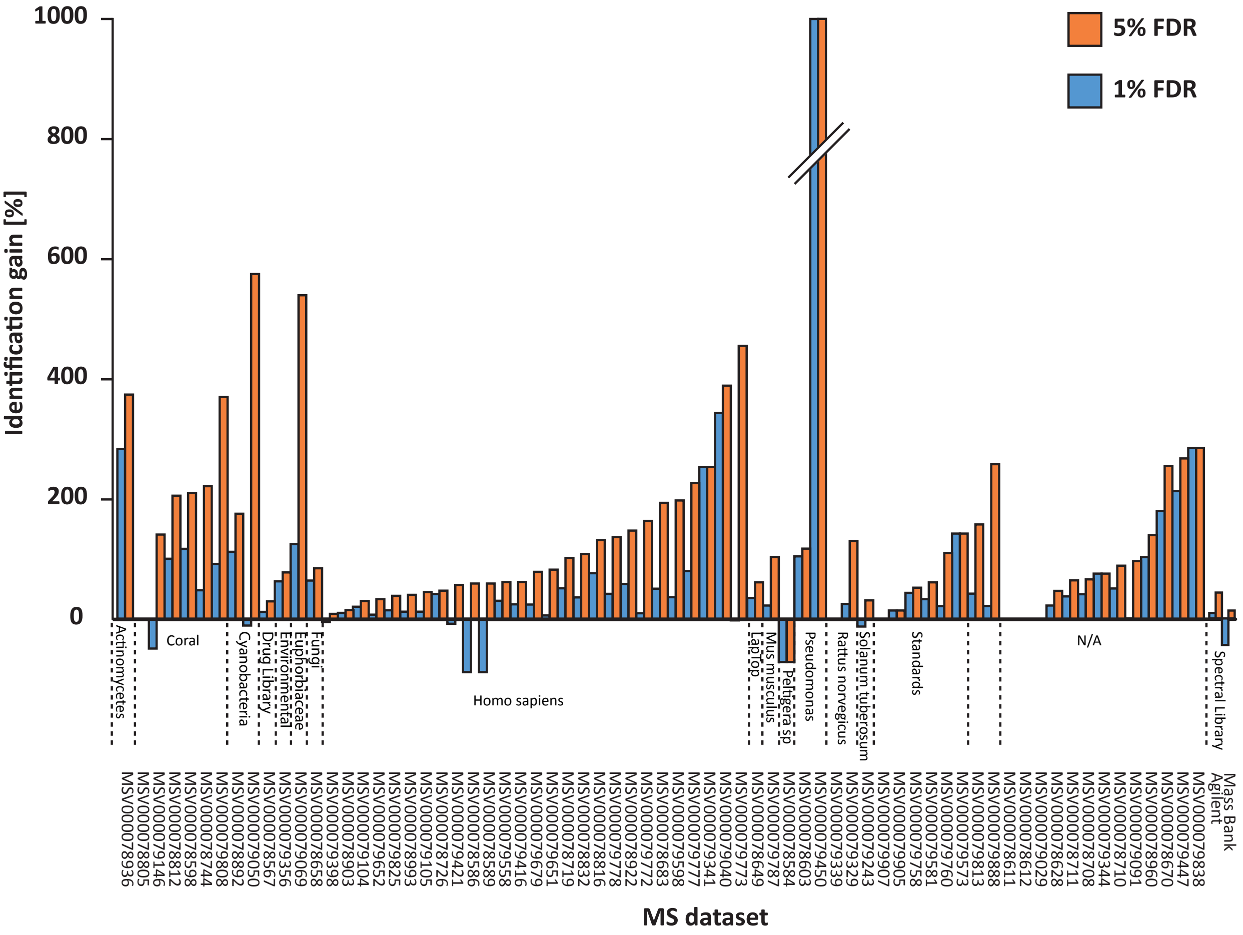

Supplementary Figure 7: FDR based annotations for metabolomics 70 projects from human, microbes, plants, marine-organism and other derived metabolomics data. The plot shows the percent gain in annotations for each of the data sets in GNPS-MassIVE at 1% and 5% FDR in relationship to the sample characteristics (organism etc).

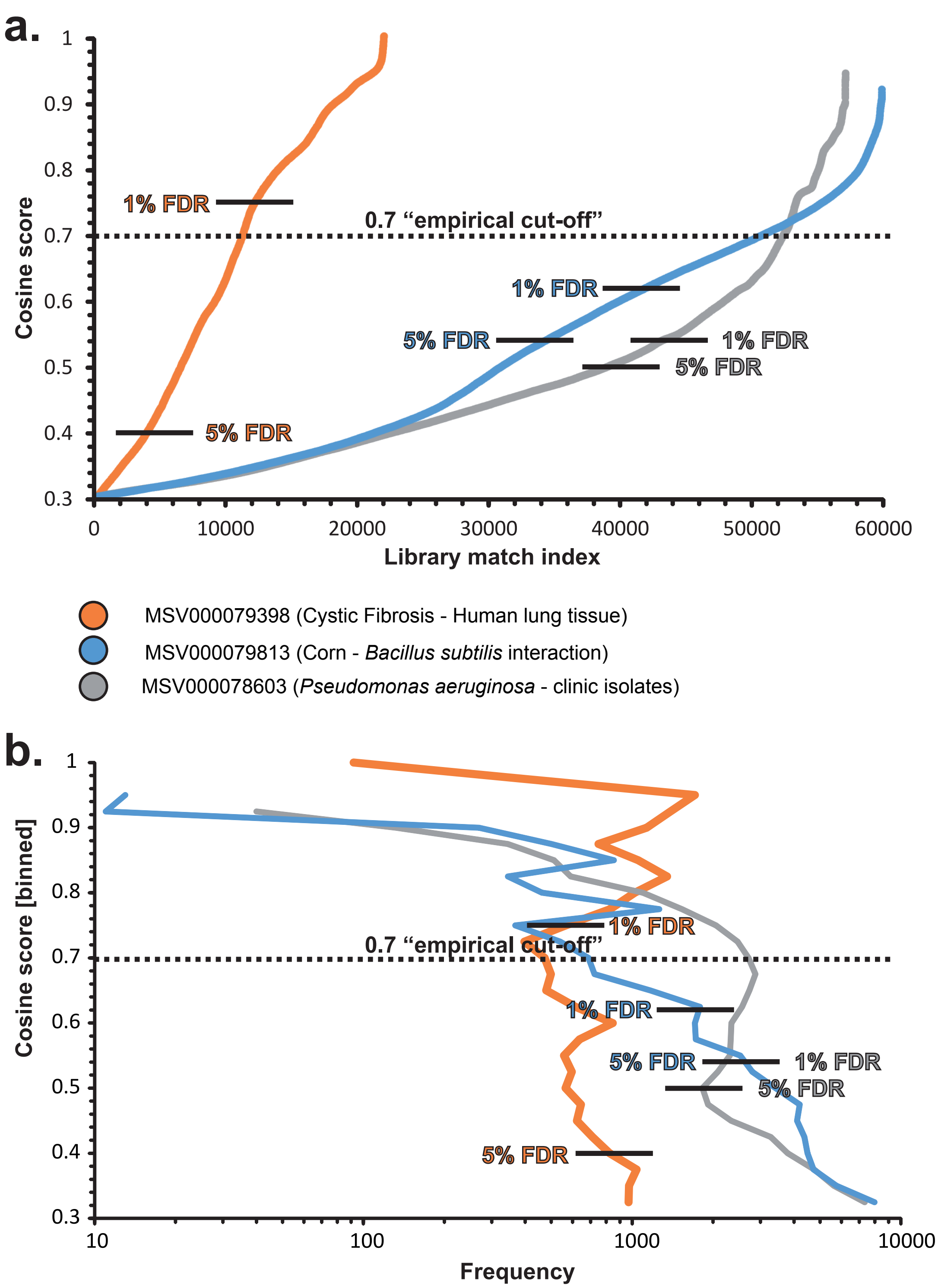

Supplementary Figure 8: Spectral matches (or match rate) vs. score, at a minimum of 6 ions matching. a. Accumulative library index matches at a given cosine score vs. cosine match. b. Frequency of spectral matches (binned in 0.025 cosine units) vs. cosine score.

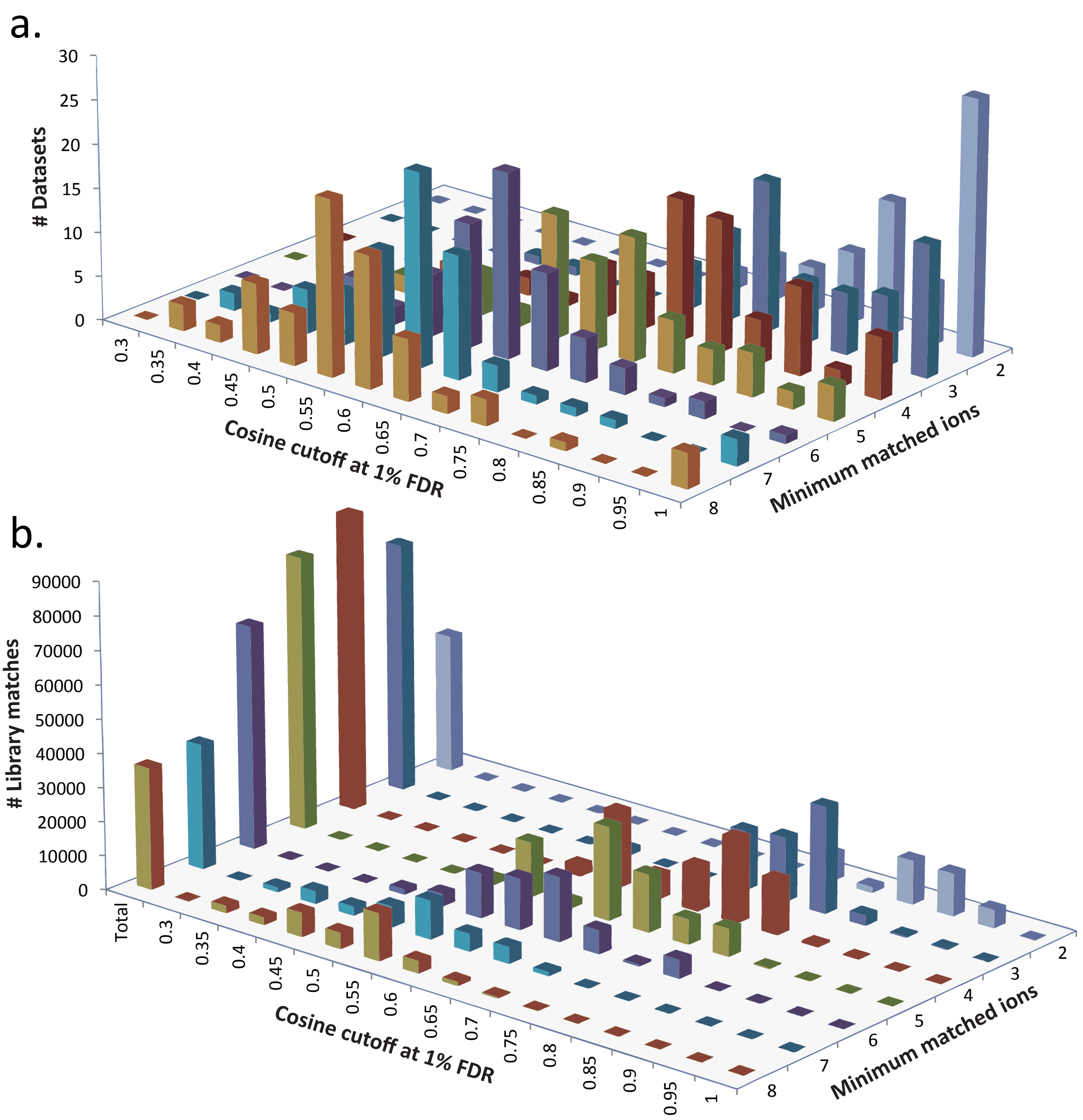

Supplementary Figure 9: 3D visualization of the impact of number of matching fragment ions in a spectrum and cosine score at 1% FDR. a. Frequency of datasets in relationship to number of minimum fragment ions to match and cosine at 1% FDR estimation. b. The number of MS/MS matches in relation to minimum matched fragment ions and cosine.

Supplementary Methods

Empirical Bayes Approach

We model the distribution of database search scores as a two-component mixture of distributions representing true positive (TP, f_{TP}) and false positive (FP, f_{FP}) identifications. Given these distributions, the Posterior Error Probability (PEP) estimate is the conditional probability that an observed hit with score x is incorrect,

$$\widehat{PEP}(x) = \frac{f_{\text{FP}}(x)}{f_{\text{FP}}(x) + f_{\text{TP}}(x)}.$$

To compute the FDR for a certain score threshold x_s, we estimate the expected value of the fraction of incorrect hits among all hits (correct or incorrect) with score at least x_s (*Expected Bayes* approach). Let $S = \{x_1, x_2, ..., x_m\}$ be the set of match scores $\geq x_s$. The probability $P(X = k)$ is the probability that exactly k of these m scores are from incorrect hits. Then the expected FDR is

$$\widehat{FDR}(x_s) = \sum_{i=0}^{m} P(X = i) \cdot \frac{i}{m} = \frac{\mathbb{E}[X]}{m}.$$

The probability of having k "successful" trials out of a total of m trials is described by a Poisson binomial distribution, a discrete probability distribution of a sum of independent Bernoulli trials that are not necessarily identically distributed. The expected value of a random variable X that is Poisson binomial distributed with "success" probabilities $\widehat{PEP}(x_1)$, $\widehat{PEP}(x_2)$, $\ldots$, $\widehat{PEP}(x_m)$ is $\mathbb{E}[X] = \sum_{i=1}^{m} \widehat{PEP}(x_i)$ and, thus, the expected FDR reduces to the average PEP of all hits with score at least x_s:

$$\widehat{FDR}(x_s) = \frac{\sum_{i=1}^{m} \widehat{PEP}(x_i)}{m}.$$

Estimating the FDR using the integral of TP and FP distributions resulted in slightly worse estimates.

For the distribution of TP and FP scores, we tested six parametric distributions and their mirrored counterparts, namely Gamma, Gumbel, Weibull, Exponential, Normal and Logistic distribution. We mirror distributions around the y-axis and shift them by $x_{\max}$. To avoid infinity probability density values for zero entries, we shift functions by -10^{-5}. To find the best model, each distribution is fitted individually using Maximum Likelihood estimates, and evaluated based on the resulting likelihood. We find that scores of FPs are modeled best using a Gamma distribution, and scores of TPs using either a mirrored Gamma distribution, a mirrored Gumbel distribution, or a mirrored Weibull distribution.

In application, we do not know which scores belong to correct and which to bogus hits. To this end, we use Expectation Maximization (EM) to find the best-fitting mixture of distributions according to likelihood. We consider three combinations of parametric distributions for FP/TP, namely Gamma/Gamma (mirrored), Gamma/Gumbel (mirrored) and Gamma/Weibull (mirrored). Fitted distributions for FPs and TPs closely match the histograms for the Agilent and MassBank datasets, although it is not known to the EM algorithm which hits are true and which ones are false. In most cases, the best-fitting distributions of the two-component mixture correspond to the two individually determined distributions for TP and FP scores. We note that shapes of the available distributions for TPs (mirrored Gamma, Gumbel and Weibull distribution) are very similar.

In our evaluations using the Agilent and MassBank datasets, distributions were fitted using Expectation Maximization. For the unfiltered Agilent dataset, EM determined that a mirrored Weibull distribution for TP and a Gamma distribution for FP best fitted the joint distribution. For the unfiltered MassBank dataset, EM determined a mirrored Gumbel distribution for TP and a Gamma distribution for FP. For both noise-filtered datasets, EM determined a mirrored Gamma distribution for TP and a Gamma distribution for FP.
