# Supplementary material for: Significance estimation for large scale metabolomics annotations by spectral matching
Source: Nat Commun. 2017 Nov 14;8:1494. doi: 10.1038/s41467-017-01318-5 (PMC5684233; doi:10.1038/s41467-017-01318-5)
Supplement: Supplementary file 2 — Peer Review File [file 41467_2017_1318_MOESM2_ESM.pdf]

Editorial Note: this manuscript has been previously reviewed at another journal that is not operating a transparent peer review scheme. This document only contains reviewer comments and rebuttal letters for versions considered at Nature Communications.
